# Spanked

**Published:** 1879-04

**Authors:** 


Spanked.—The Troy *Times* has heard of an astonished doctor out West, who was called to a critical case, but found his "occupation gone." Presence of mind and rough determination proved as effectual in a dangerous emergency as mere skill. The *Times* tells how it was:

A small yearling youngster at Fort Wayne, Indiana, had the misfortune to suck a kernel of corn into its windpipe the other day. The doctor was sent for in haste, and announced that it would be necessary to perform the operation of tracheotomy to save the child's life. The Hoosier mother, familiar with a practice of domestic surgery of a different sort, and not pleased with the idea of having the child's windpipe cut open, seized the sufferer by one leg, and holding him up, head downward, administered sundry resounding spanks. There was a sound not unlike the report of a pop-gun, and the kernel of corn was ejected with great force. The child was at once relieved, and recovered, of course.

The astonished physician declared that, for a "*corn*-doctor," this Hoosier mother beat him all hollow.—*The Sanitarian.*

